# A clinical investigation using reflectance confocal microscopy to evaluate the add-on efficacy of the Shumin treatment instrument combined with doxycycline for rosacea

**DOI:** 10.3389/fmed.2026.1826076

**Published:** 2026-05-25

**Authors:** Yimin He, Jing Yuan, Mengting Hu, Hui Wan, Ying Gao

**Affiliations:** Department of Dermatology, The Central Hospital of Wuhan, Tongji Medical College, Huazhong University of Science and Technology, Wuhan, China

**Keywords:** demodex mites, doxycycline, reflectance confocal microscopy, rosacea, Shumin treatment instrument

## Abstract

**Objective:**

This study aimed to compare the clinical efficacy and safety of two rosacea treatment regimens: oral doxycycline plus the Shumin treatment instrument and oral doxycycline plus red light therapy. Objective assessments, including reflectance confocal microscopy, were used to evaluate both regimens.

**Materials and methods:**

A total of 60 patients with rosacea were randomly divided into two groups, each containing 30 patients. The control group received doxycycline (100 mg/day) orally combined with red light therapy for 12 weeks (twice a week), whereas the experimental group received doxycycline (100 mg/day) orally combined with Shumin treatment instrument therapy for 12 weeks (twice a week).

**Results:**

The efficacy rate was 93.3% in the experimental group and 80.0% in the control group. Following treatment, significant reductions in Dermatology Life Quality Index scores and Demodex counts were achieved in both groups (all *p* < 0.001). However, the experimental group exhibited superior improvements in Dermatology Life Quality Index scores and affected hair follicle counts compared with the control group (*p* < 0.05). No significant difference was observed in the reduction in the total *Demodex* count between the groups. Similarly, no significant difference was observed in the change in the total hair follicle number between the groups. Furthermore, the relapse rate was notably lower in the experimental group than in the control group (*p* = 0.026).

**Conclusion:**

When used as an adjunctive therapy with oral doxycycline, the Shumin device yields better clinical outcomes than red light therapy for treating rosacea and has a favorable safety profile. These preliminary results establish a basis for the use of adjuvant physical interventions for rosacea.

## 1 Introduction

Rosacea is a chronic inflammatory skin disease that primarily affects the blood vessels and pilosebaceous glands of the face. It is characterized by paroxysmal flushing, persistent erythema, telangiectasia, papules, and pustules, accompanied by symptoms such as burning, stinging, dryness, and itching. Epidemiological studies indicate that rosacea is more prevalent in people with fair skin, with a global incidence of approximately 5.5%. Rosacea commonly appears after the age of 30 years and is more prevalent in women (1). Clinically, it is divided into erythematotelangiectatic rosacea (type I), papulopustular rosacea (type II), phymatous rosacea (type III), ocular rosacea (type IV), and the granulomatous rosacea variant. Different types often overlap (2). The majority of patients have comorbid psychological problems, which cause difficulties in social interactions and work. Rosacea is difficult to treat and prone to recurrence. To further optimize therapeutic regimens and seek safer, more effective combined treatment strategies, this study compared the efficacy and safety of two regimens: oral doxycycline combined with the Shumin treatment instrument and oral doxycycline combined with red light therapy. The results were satisfactory.

## 2 Materials and methods

### 2.1 Clinical data

With informed consent obtained from all patients and approval from the hospital ethics committee (Approval No. WHZXKYL2022-185), 60 patients with rosacea were recruited from our outpatient department between September 2022 and August 2023. A random number table was used to generate a random sequence, and patients were randomly assigned to the experimental group or the control group in a 1:1 ratio. Allocation concealment was implemented using sequentially numbered, sealed, opaque envelopes. Due to the different physical treatments applied, blinding of the patients and operators was not feasible. The control group included 12 men and 18 women aged between 18 and 60 years. In the experimental group, there were 11 men and 19 women aged between 18 and 60 years (Table 1). All participants had Fitzpatrick skin types III–IV. All patients included in the study had erythematous or papulopustular rosacea. The duration of the disease ranged from 3 months to 6 years. All 60 patients completed the treatment and follow-up, with no dropouts.

**Table 1:** Gender and age distribution (‾*x ± s*).

Group	Number	Male	Female	*χ^2^*	*p*	Age	*χ^2^*	*p*
Experimental group	30	11	19	0.071	0.791	33.300 ± 9.380	0.408	0.982
Control group	30	12	18			32.830 ± 8.860		

### 2.2 Inclusion criteria

Patients were diagnosed with rosacea according to the diagnostic criteria specified in the 2021 Chinese Rosacea Diagnosis and Treatment Guideline (3). Within the previous 4 weeks, no tretinoin, antibiotics, or other relevant drugs had been administered, and within the past 12 weeks, no photoelectric treatment had been received. Patients were between 18 and 60 years of age, without gender restriction. Additionally, patients had no severe systemic diseases. Pregnant women, lactating women, and those with recent fertility requirements were excluded.

### 2.3 Methods

#### 2.3.1 Interventions and blinded outcome assessment

All patients received 100 mg/day of doxycycline orally for 12 weeks. In the control group, Avene Tolerance Extreme emollient cream was applied to their face, followed by red light irradiation for 20 min twice a week for 12 weeks. In the experimental group, the cream was applied using a Shumin treatment instrument twice a week for 12 weeks. During the treatment, patients were required to follow a light diet and practice sun protection. Two trained, independent dermatologists performed all outcome assessments. The dermatologists were blinded to the group assignment. Any disagreements between the two assessors were resolved by a third, senior dermatologist who was also unaware of the group allocation.

#### 2.3.2 Shumin treatment instrument treatment

The Derma-CR model Shumin treatment instrument (Erlang Chuangye Road, Erlang Sub-district, Jiulongpo District, Chongqing, China) was used in high-frequency, alternating electromagnetic wave mode with a pulse frequency of 4 MHz. The device has 15 adjustable energy levels, and levels 2–4 were used in this study based on skin response and patient comfort. The handle and the introduction head were disinfected with 75% alcohol. After facial cleansing, the patients were asked to lie in a supine position. The treatment started with a low energy setting, which was adjusted (levels 2–4) according to skin temperature and patients’ feedback. This device enhances the transdermal absorption of topical emollients while in use. Avene Tolerance Extreme emollient cream was applied to the patients’ faces, and the operator made circular motions evenly from the bottom to the top and from the middle of the face to the front of the ears. The water outlet control button could be used to replenish water at any time. The process was repeated 3–4 times for a total treatment duration of 20 min per session. After the treatment, faces were cleaned with warm water, and Avene Tolerance Extreme emollient cream was reapplied. All treatment sessions were performed by the same experienced operator. Each treatment was administered twice weekly for 12 consecutive weeks.

#### 2.3.3 Red light treatment

Red light irradiation was performed using a therapeutic device (Kenuo Building, Xuzhou Economic Development Zone, Jiangsu Province, China) with a wavelength of 633 nm and a power density of 33 mW/cm^2^. The distance from the device to the patients’ faces was approximately 6 ± 1 cm. Each 20-min session was administered twice weekly for 12 consecutive weeks. All procedures were performed by the same experienced operator to ensure consistency.

#### 2.3.4 Reflectance confocal microscopy (RCM) detection

Before the examination, the facial skin was thoroughly cleaned, and identical facial regions were selected for RCM detection using the VivaScope 1,500 (50 Methodist Hill Dr Ste 1000, Rochester, NY, USA) at baseline and after treatment. RCM examinations were performed by the same experienced operator who was blinded to the patient group allocation. A fixed target lesion was selected for RCM scanning in each patient, and its location was documented using clinical photography at each visit to ensure accurate repositioning. Using water as the medium, a disposable patch was placed on the skin lesion. Scanning was performed from the top to the bottom. When a clear image of *Demodex folliculorum* was observed, a 2 mm × 2 mm area was scanned horizontally. The area of a single image was 0.5 mm × 0.5 mm. A total of three fields of view were evaluated for each patient at the same target lesion. The average Demodex counts, affected follicle counts, and total follicle counts from these three fields were used for subsequent statistical analyses. Two trained dermatologists, who were blinded to the treatment group and time point, independently assessed all RCM images. Any discrepancies were resolved by consensus with a third senior blinded dermatologist to minimize observer bias.

#### 2.3.5 Study endpoints

The primary endpoint was defined as clinical efficacy, as evaluated by the Physician’s Global Assessment (PGA). The secondary endpoints included changes in the Dermatology Life Quality Index (DLQI) score, Demodex counts, affected follicle counts, total follicle counts, recurrence rate, and safety outcomes.

#### 2.3.6 Standardized clinical photography

All clinical photographs were taken at baseline and after treatment. A fixed digital camera was used under consistent indoor lighting and with consistent camera distance, angle, and patient head positioning to ensure the images were standardized and comparable.

### 2.4 Efficacy evaluation criteria

#### 2.4.1 Clinical efficacy

Clinical efficacy was evaluated according to the PGA for rosacea. The evaluation was as follows: cure: 100% regression of skin lesions; marked effect: 75%–99% regression of skin lesions; effective: 50–74% regression of skin lesions; ineffective: <50% regression of skin lesions or aggravation. The effective rate was calculated as follows: (number of cured cases + number of markedly effective cases + number of effective cases)/total number of cases × 100% (4).

#### 2.4.2 DLQI

The DLQI was completed by the patients before and after treatment. Scores were assigned from 0 to 3 according to the following categories: no impact, mild impact, moderate impact, and severe impact. Impact scores were categorized as follows: no impact: 0–5 points; mild impact: 6–10 points; moderate impact: 11–20 points; and severe impact: 21–30 points (5).

#### 2.4.3 RCM image index observation and counting

The total number of hair follicles, the number of affected hair follicles, the number of *Demodex folliculorum* in a single hair follicle, and the total number of *Demodex folliculorum* in the 2 mm × 2 mm area of the skin lesions were counted on the RCM images before and after treatment.

#### 2.4.4 Safety

Any adverse reactions that occurred during the treatment were strictly recorded, including dizziness, nausea, redness and swelling, stinging, itching, and burning. The time of onset, severity, and outcome of the adverse reactions were recorded.

### 2.5 Follow-up

All patients were followed up once every 2 weeks until 4 weeks after the end of the treatment. Five patients experienced dizziness and nausea after oral doxycycline administration; however, they could tolerate these symptoms. No obvious adverse reactions were observed during red light irradiation or Shumin treatment. Recurrence was defined as the reappearance of significant erythema, telangiectasia, papules, or pustules requiring additional treatment within 4 weeks after the end of the treatment. At 4 weeks after treatment completion, one case relapsed in the experimental group and eight cases relapsed in the control group.

### 2.6 Statistical analysis

The data were analyzed using SPSS 19.0 statistical software. The ordinal PGA efficacy grades were compared between the groups using the Mann–Whitney *U*-test. The “effective rate” was reported only as a descriptive indicator and was not subjected to statistical testing. The data were not normally distributed for DLQI scores, total number of hair follicles, number of affected hair follicles, and total Demodex counts. Within-group comparisons of pre-treatment and post-treatment values were performed using the Wilcoxon signed-rank test. Improvement magnitude (calculated as baseline value minus post-treatment value) was compared between the groups using the Mann–Whitney *U*-test. All tests were two-tailed, and a *p*-value of <0.05 was considered statistically significant.

## 3 Results

### 3.1 Comparison of clinical efficacy

**Figure 1:**
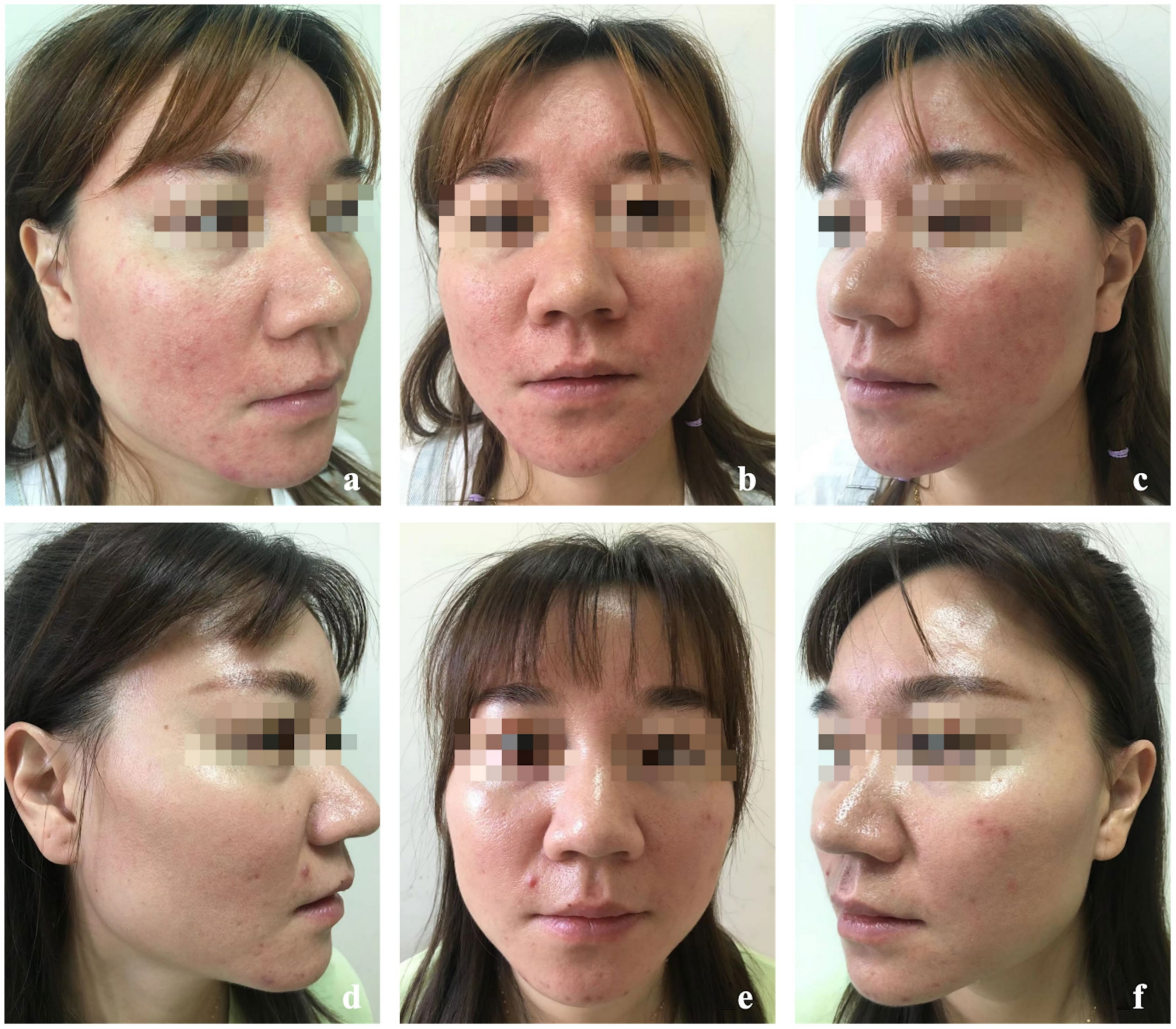
Clinical images of the experimental group before and after the treatment. **(a–c)** Bright red erythema was visible on the face before the treatment. **(d–f)** The erythema significantly subsided after the treatment.

The efficacy rate of the experimental group was 93.3%, which was significantly higher than that of the control group (80.0%). A significant difference was observed in the distribution of PGA efficacy grades between the two groups (*Z* = −2.59, *p* = 0.010), with the experimental group showing a significantly better ordinal rank of efficacy than the control group (Figure 1 and Table 2).

**Table 2:** Comparison of clinical efficacy.

Group	Number	Cured	Markedly effective	Effective	Ineffective
Experimental group	30	7	19	2	2
Control group	30	4	11	9	6
*Z*	−2.590				
*p*	0.010				

### 3.2 Comparison of DLQI scores

In both groups, DLQI scores decreased significantly after treatment (Wilcoxon signed-rank test, both *p* < 0.001). The magnitude of DLQI improvement was significantly higher in the experimental group than in the control group (*Z* = −2.171, *p* = 0.030) (Table 3).

**Table 3:** Comparison of DLQI scores [M(Q_25_, Q_75_)].

Group	Number	Before treatment	After treatment	*Z*	*p*
Experimental group	30	20.500 (20.000, 21.000)	3.000 (3.000, 3.000)	−4.787	< 0.001
Control group	30	19.500 (19.000, 20.000)	5.000 (5.000, 5.000)	−4.627	< 0.001
*Z*	−2.171				
*p*	0.030				

### 3.3 Comparison of the number of *Demodex folliculorum*

**Figure 2:**
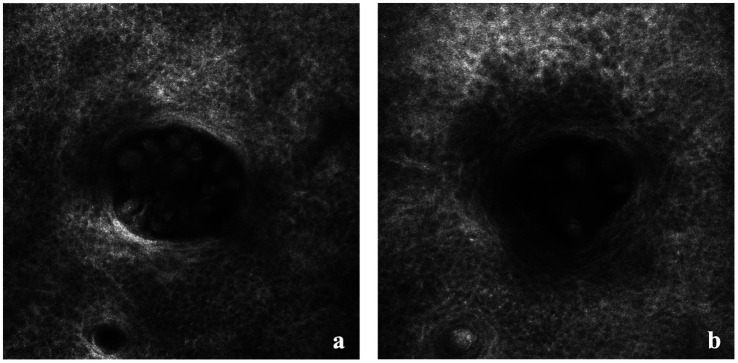
RCM images of the experimental group before and after the treatment. **(a)** Before the treatment, the hair follicle openings were dilated, and multiple clustered roundish grayish-white structures were visible. **(b)** After the treatment, the hair follicle openings shrank, and the number of *Demodex folliculorum* decreased.

*Demodex folliculorum* were detected in all 60 patients. Under RCM, cross-sections showed clustered, round-like, gray-white structures in the enlarged hair follicle infundibulum, and there could be up to 14 of these structures in a single hair follicle. After the treatment, the hair follicles became smaller than before, and the number of *Demodex folliculorum* decreased (Figure 2). No significant changes were observed in the total number of hair follicles before or after the treatment in either group, and no significant difference was observed in the extent of change between the two groups (all *p* > 0.05). Both groups showed a significant reduction in the number of affected hair follicles and total Demodex counts after the treatment (all *p* < 0.001). However, the experimental group had a significantly greater reduction in affected hair follicles than in the control group (*Z* = −2.415, *p* = 0.016), while the reduction in the total Demodex counts did not differ significantly between the two groups (*Z* = −0.112, *p* = 0.911) (Table 4).

**Table 4:** Comparison of the total number of hair follicles, the number of affected hair follicles, and the total number of demodex folliculorum before and after treatment [M(Q_25_, Q_75_)].

Evaluation indicators	Experimental group			Control group			*Z*	*p*
	Before treatment	After treatment	*p*	Before treatment	After treatment	*p*		
Total number of hair follicles	17.000 (15.000, 20.000)	17.500 (15.000, 19.000)	0.330	18.000 (17.000, 19.000)	17.000 (17.000, 18.000)	0.388	−0.742	0.458
Number of affected hair follicles	6.000 (6.000, 6.000)	5.000 (4.000, 5.000)	<0.001	5.500(5.000, 9.000)	2.500 (2.000, 5.000)	<0.001	−2.415	0.016
Total number of demodex folliculorum	13.000 (13.000, 16.000)	9.5000 (9.000, 11.000)	<0.001	11.500 (11.000, 17.000)	10.000 (10.000, 11.000)	<0.001	−0.112	0.911

### 3.4 Comparison of relapse rates

The relapse rate in the experimental group was 3.33% (1/30), and that in the control group was 13.33% (8/30). Fisher’s exact test revealed a significant between-group difference in recurrence rates (*p* = 0.026).

## 4 Discussion

The exact etiology and pathogenesis of rosacea are currently unclear. Possible causes include neurovascular disorders, immune disorders, barrier dysfunction, genetic factors, and microbial infections. Rosacea generally does not heal on its own and is difficult to treat and prone to recurrence. Various treatment methods target different aspects of its pathogenesis. These methods include basic care such as cold therapy; topical treatments such as ivermectin, metronidazole, azelaic acid, and benzoyl peroxide; systemic treatments such as antibiotics, isotretinoin, hydroxychloroquine, and beta-blockers; biological agents, such as Janus kinase (JAK) inhibitors; physical treatments such as intense pulsed light, pulsed dye lasers, and photodynamic therapy; and surgical treatments (6, 7).

In recent years, the relationship between the skin barrier and rosacea has received increasing attention. Skin barrier dysfunction is not only a clinical manifestation but also a contributing factor. Dysfunction in skin permeability and imbalance in the microenvironment, such as sebum, pH level, epidermal water content, and transepidermal water loss, make the skin more sensitive to external stimuli and promote microbial colonization, which can trigger the disease. Therefore, in addition to avoiding triggers, it is necessary to strengthen cold compresses and moisturization, reduce transepidermal water loss by locking in moisture, and promote barrier repair (8). The principle of Shumin treatment is to stimulate collagen regeneration by heating with high-frequency alternating electromagnetic waves; this enhances skin tolerance, promotes blood circulation, promotes the metabolism of inflammatory factors by releasing reactive oxygen species, repairs the skin barrier, achieves rapid water replenishment and water-locking, and promotes oil–water balance.

There is ongoing controversy both in China and internationally regarding the relationship between microorganisms and rosacea, with a majority of the research focused on *Demodex folliculorum*. RCM studies have shown that the density of *Demodex folliculorum* in patients with rosacea increases significantly. Rosacea is conducive to the proliferation of *Demodex folliculorum*, and the over-proliferated *Demodex folliculorum* causes an immune response, aggravating rosacea and forming a “vicious cycle” (9). The present study has found that neither treatment significantly altered the total number of hair follicles, indicating favorable safety profiles and the absence of irreversible follicular damage. Both regimens significantly reduced the number of affected hair follicles and total *Demodex folliculorum* counts, confirming their efficacy in improving the inflammatory status associated with *Demodex* infestations. However, the experimental group showed a significantly greater reduction in affected hair follicles than the control group. These benefits may be related to the anti-inflammatory and microenvironment-modulating effects of the Shumin treatment. Notably, the reduction in total *Demodex folliculorum* counts was comparable between the two groups, suggesting similar direct anti-parasitic efficacy. The experimental group’s advantage appeared to be more pronounced in repairing the affected follicles and improving inflammation. Further studies with larger sample sizes and longer follow-ups are needed to confirm whether these results lead to more sustained clinical outcomes and lower recurrence rates. Doxycycline, as a semi-tetracycline-class broad-spectrum antibiotic, has the advantages of easy absorption, a long half-life, and low drug resistance. In addition, it also has anti-parasitic, anti-inflammatory, anti-oxidative, neuroprotective, immunomodulatory, and angiogenesis-inhibiting properties; therefore, it is effective in the treatment of rosacea (10). Nearly all patients have varying degrees of skin barrier dysfunction. When used as a complementary adjuvant therapy with oral doxycycline, the Shumin device helps optimize clinical outcomes, improve skin barrier damage, and shorten disease duration to a certain extent compared with conventional red light intervention. It also presents a favorable safety profile, with no severe adverse events, and demonstrates good patient compliance. However, this study has several limitations. Due to the administration of doxycycline to all patients and the lack of a control group that received only one treatment, the independent efficacy of the Shumin device could not be adequately assessed. Moreover, the 4-week post-treatment follow-up period was too short to fully evaluate the long-term prognosis and recurrence of this chronic, relapsing condition. As a single-center trial with limited baseline data collection, the study’s findings may be difficult to generalize to other populations. Additionally, despite an independent RCM evaluation, subtle observer bias in image interpretation cannot be entirely excluded. Finally, the relatively small sample size is a limitation of this exploratory study.

In conclusion, the treatment of rosacea requires an individualized chronic disease management model. Based on appropriate basic care, it is necessary to combine drug and physical treatments and develop individualized plans for different patients and disease subtypes. The combination of oral doxycycline and Shumin treatment (a dual-combination regimen) shows preliminary superior therapeutic effects compared to doxycycline combined with red light therapy. This combination may help further improve clinical outcomes, reduce adverse drug reactions, relieve symptoms of skin sensitivity, and potentially shorten the disease course. However, given the limitations of this single-center, small-sample, and short-term follow-up study, the current findings are preliminary. Further large-sample and multicenter studies are needed for verification before widespread clinical application.

## Data Availability

The original contributions presented in the study are included in the article/supplementary material, further inquiries can be directed to the corresponding authors.
